# Effects of miglustat therapy on neurological disorder and survival in early-infantile Niemann-Pick disease type C: a national French retrospective study

**DOI:** 10.1186/s13023-023-02804-4

**Published:** 2023-07-21

**Authors:** Cécile Freihuber, Bahia Dahmani-Rabehi, Anaïs Brassier, Pierre Broué, Claude Cances, Brigitte Chabrol, Didier Eyer, François Labarthe, Philippe Latour, Thierry Levade, Samia Pichard, Caroline Sevin, Marie T. Vanier, Bénédicte Héron

**Affiliations:** 1 Department of Paediatric Neurology, Reference Centre for Lysosomal Diseases, Armand Trousseau-La Roche Guyon Hospital and Hospital-University I2-D2 Federation, Sorbonne-Université, Paris, France; 2 Department of Paediatrics, Jean Verdier University Hospital, Bondy, France; 3 Department of Metabolic Disorders, Reference Center for Inborn Errors of Metabolism, Necker-Enfants Malades University Hospital, Paris, France; 4 Department of Paediatric Hepatology and Metabolic Disorders, Reference Centre for Inborn Errors of Metabolism and Genetic Cholestasis, Children’s Hospital Toulouse University Hospitals, Toulouse, France; 5 Department of Paediatric Neurology, Purpan University Hospital, Toulouse, France; 6 Department of Paediatric Neurometabolism, La Timone University Hospital, Marseille, France; 7 Department of Paediatrics, Haguenau Hospital, Hagueneau, France; 8 CRMR ToTeM, Department of Pediatrics, Hôpital Clocheville, CHRU Tours, and Laboratoire N2C, Inserm U1069, Université François Rabelais de Tours, 37 000 Tours, France; 9 Department of Biochemistry and Molecular Biology, Hospices Civils de Lyon, Lyon, France; 10 INSERM U1037 (Cancer Research Centre of Toulouse), Université Paul Sabatier, Toulouse, France; 11 Department of Clinical Biochemistry, Toulouse University Hospital, Toulouse, France; 12 Department of Paediatric Neurology, Kremlin-Bicêtre University Hospital, Paris, France; 13 Laboratoire Gillet-Mérieux, Lyon-East University Hospital, Hospices Civils de Lyon, Lyon, France; 14 INSERM U820, Lyon, France

**Keywords:** Niemann-Pick disease type C, Paediatric, Early infantile, Miglustat, Efficacy, Survival, Safety, France

## Abstract

**Background:**

Niemann-Pick disease type C (NP-C) is a rare neurovisceral lysosomal lipid storage disease characterized by progressive neurodegeneration and premature death. While miglustat can stabilize neurological manifestations in later onset forms of NP-C, its efficacy in the early-infantile neurological form has not been demonstrated. In this observational retrospective study, we compared long-term neurodevelopmental outcome and survival between an untreated and a treated group of early infantile NP-C patients.

**Methods:**

Data available on all NP-C patients with early infantile neurological onset diagnosed in France between 1990 and 2013 were compiled. Patients with incomplete data or who had died from a systemic perinatal, rapidly fatal form were excluded.

**Results:**

Ten patients were included in the treated group (year of birth: 2006–2012), and 16 patients in the untreated group [born 1987–2005 (n = 15), 2012 (n = 1)]. The median age at neurological onset was 9 months (5–18) in the treated group, and 12 months (3–18) in the untreated group (p = 0.22). Miglustat therapy was started at a median age of 24.5 months (9–29) and median duration was 30 months (11–56). Gastrointestinal adverse events were reported in 7/10 patients on miglustat. All patients developed loss of psychomotor acquisitions or additional neurological symptoms despite miglustat therapy. The ages of developmental milestones and neurological involvement did not significantly differ between the two groups. Four patients in the untreated group were lost to follow up. The 22 remaining patients had died by the end of the study and no patient survived beyond the age of 7.4 years. The median survival age was 4.42 years in the untreated group and 5.56 years in the treated group; the Kaplan–Meier survival curves were not significantly different (log-rank test: p = 0.11).

**Conclusions:**

Miglustat allowed no significant long-term neurodevelopmental improvement nor significant increase of survival in patients with early infantile NP-C.

**Supplementary Information:**

The online version contains supplementary material available at 10.1186/s13023-023-02804-4.

## Background

Niemann-Pick disease type C (NP-C) is a rare autosomal recessive neurovisceral lipid storage disease characterized in a majority of cases by progressive neurological deterioration leading to premature death, and (hepato)splenomegaly; its estimated incidence appears close to 1:100,000 live births [1–3]. NP-C is caused by bi-allelic pathogenic variants in either the *NPC1* (in 95% of cases) or the *NPC2* gene (in around 5% of cases) [1], leading to impaired intracellular lipid trafficking and accumulation of cholesterol and sphingolipids in the brain and other tissues [4].

Visceral symptoms include neonatal cholestasis, hepatosplenomegaly and pulmonary dysfunction. Liver failure causes rapid death (before 3–6 months of age) in 5–10% of neonates presenting with a cholestatic icterus [1, 5]); severe pulmonary insufficiency is lethal in a few others. Besides this rare systemic perinatal rapidly fatal form, four main clinical forms of NP-C are described according to the age of onset of neurological symptoms: early-infantile (EI) (< 2 years), late-infantile (2–5 years), juvenile (6–14 years), and adolescent-adult (≥ 15 years) [1, 6, 7]. A few additional patients with systemic disease only [8, 9] are temporarily unclassifiable since most of them—if not all—will later on enter one of the neurological forms. Typical neurological findings in paediatric NP-C include delay in psychomotor milestones, vertical supranuclear gaze palsy (VSGP), ataxia, dystonia, dysarthria, dysphagia, gelastic cataplexy, seizures and psychomotor or cognitive deterioration [7, 10]. These manifestations have a continuous, unbroken progression, however consistently faster in patients with onset in early childhood compared with those showing a later onset form [6].

At the time of our study, miglustat was the only disease-specific therapy available for the treatment of progressive neurological manifestations in patients with NP-C; to-date, it remains the only approved drug. This small iminosugar molecule able to partially cross the blood–brain barrier is a competitive inhibitor of glucosylceramide synthase, which catalyses the first committed step in the synthesis of glycosphingolipids accumulating in NP-C, but its mode of action appears more complex [11]. Particularly, concomitant inhibition of the GBA2 glucosylceramidase might be involved [12].

The approval of miglustat was based on data from a randomized clinical trial [13], long-term extension studies [14, 15] and a retrospective observational cohort study [16], demonstrating stabilization of key neurological manifestations. A comprehensive review of published studies of miglustat in NP-C has been published [11], as well as a consensus clinical management guideline [3].

While miglustat can stabilize neurological manifestations in later onset forms of NP-C, its efficacy in the EI form has not been demonstrated [17–19] 11/07/2023 21:09:00. Most published studies on miglustat efficacy included only a small number of EI-NP-C patients, with no long-term evaluation of therapy (duration of treatment < 2 years). The present observational retrospective multicentric study on patients with NP-C from the French cohort is the first one to specifically focus on the EI-NP-C form. We aimed to compare long-term neurodevelopmental outcome and survival between a miglustat-treated group and an untreated group.

## Methods

### Patients and study design

All children from the French NP-C cohort with a neurological onset before 2 years of age who had been diagnosed between April 1990 and June 2013 (i.e., before the introduction of specific plasma biomarkers) were included. Five additional early infantile patients diagnosed later (2014–2017) had an onset of neurological symptoms at around 18 months of age. However, they were not analysed in the present study since, besides receiving miglustat, they were included in therapeutic trials (studying oral arimoclomol or intrathecal cyclodextrin), or treated with arimoclomol in an early access programme. The date of diagnosis was defined as the date of skin biopsy for filipin test or sample for gene sequencing analysis if done first. Patient U5 (sister of U14) had been diagnosed prenatally. Patients had been referred to the "*CRML*" (Reference Centre for Lysosomal Diseases) at the paediatric Trousseau University Hospital site, or were identified through the laboratory which centralized requests for biochemical and genetic testing of NP-C, in collaboration with the National Committee for Evaluation of Treatment of Neurolipidoses "*CETNL*". This methodology has allowed optimal exhaustivity for the French territory. For all included patients, the diagnosis was confirmed by filipin test [20, 21] and/or genetic testing (Table 1). All available medical files from patients with NP-C were collected between August 2015 and December 2021. Recorded data were anonymised and pooled for analysis. All information was accessed in accordance with applicable laws and ethical requirements for the study period concerned. Preliminary results were presented in short abstract form [22].

**Table 1:** Biochemical and genetic testing for NP-C diagnosis

Patient	Gender	Age at diagnosis	Mutated gene	*NPC1 or NPC2* gene variants	Filipin test
U1	M	2 y	*NPC1*	p.(Asp1097Asn)/p.(Leu1248fs)	Classic
U2	F	6 m	*NPC1*	p.(Gly1240Arg)/p.(Gly1240Arg) ♦	Classic
U3	M	21 m	*NPC1*	p.(Gly1240Arg)/p.(Gly1240Arg) ♦	Classic
U4	F	2 y	*NPC1*	p.(Pro543Leu)/p.(Gly319fs)	Classic
U5^§^♣♣	F	ante-natal	*NPC1*	p.(Gly994fs)/p.(Gly994fs)	Classic^†^
U6	M	2 y 5 m	*NPC1*	p.(Pro377fs)/p.(Ser970fs)	Classic
U7^§^♣	M	1 d	*NPC1*	c.3246-2_3247del/c.3246-2_3247del	Not done
U8	M	4 y 7 m	*NPC1*	p.(Cys109del)/c.632-2_642del13insT	Classic
U9^§^	M	4 y 10 m	*NPC1*	p.(Arg518Gln)/p.(Arg518Gln)	Classic
U10^§^	M	3 y 3 m	*NPC1*	p.(Ala172Pro)/p.(Ala172Pro)	Classic
U11^§^	F	15 m	*NPC2*	p.(Ser67Pro)/p.(Ser67Pro)	Classic
U12	F	1 m	*NPC1*	p.(Leu724Pro)/c.3754+1G>C	Classic
U13^§^	M	2 y 10 m	*NPC2*	p.(Cys99Arg)/p.(Cys99Arg)	Classic
U14^§^♣♣	M	4 m	*NPC1*	p.(Gly994fs)/p.(Gly994fs)	Classic
U15	M	4 m	*NPC1*	p.(Trp942Cys)/p.(Gln991fs)	Classic
U16	M	2 y 9 m	*NPC1*	p.(His1239Arg)/p.(His1239Arg)	Classic
T1^§^♣	M	1 m	*NPC1*	c.3246-2_3247del/c.3246-2_3247del	Classic
T2^§^	F	14 d	*NPC1*	p.(Thr1036Met)/p.(Thr1036Met)	Classic
T3	F	3 m	*NPC1*	p.(Pro543Leu)/p.(Thr1205fs)	Classic
T4	F	5 m	*NPC1*	p.(Pro543Leu)/c.2245+1G>A	Classic
T5	F	4 m	*NPC1*	p.(Leu830Pro)/p.(Arg958*)	Classic
T6^§^	M	1 m	*NPC1*	p.(Gly1240Arg)/p.(Gly1240Arg) ♦	Classic
T7^§^	M	17 m	*NPC1*	p.(Gly1240Arg)/p.(Gly1240Arg) ♦	Classic
T8	M	4 m	NPC1	p.(Thr1205Arg)/p.(Thr1205Lys)	Classic
T9^§^	F	4 m	*NPC1*	p.(Cys63fs)/p.(Cys63fs)	Classic
T10	M	2 m	*NPC1*	p.(Thr1036Met)/p.(Thr1036Met)	Classic

U1 to U16: untreated patients; T1 to T10: miglustat-treated patients; the ♣ and ♣♣ symbols indicate siblings; § indicates consanguinity; ♦ pinpoints the four patients originating from South India or Sri Lanka, all carrying the same homozygous mutation. ^†^filipin test made on cultivated chorionic villi. Abbreviations: y = years, m = months, d = days

Patients T2, T3, T4, T5, T8, T9 correspond to cases #9, #8, #7, #2, #4, #3, respectively, in Héron et al. [10]. The highly atypical patient #6 in the latter study was excluded from the present one because in spite of showing initial neurological signs at 9 months of age, his evolution before miglustat start (when 3.5-year-old) had been minimal and similar to that seen in the late infantile form. Of note, 12 patients of the present study (2 untreated, 10 treated ones) (Additional file 1: Table S1) were included in a survey of early liver disease in NP-C [5]. Most patients in this series were also part of a wide multinational survey on survival outcome in NP-C [23].

### Neurological manifestations

The neurological manifestations were defined as milestones delay, psychomotor regression, or neurological symptoms. Age of neurological onset was determined as age of first observation by caregivers or clinicians of milestones delay and/or psychomotor regression and/or neurological symptoms. Key age milestones were established by the “*Brunet-Lézine test*”. Delayed motor development was defined by autonomous sitting position after age 9 months, standing position or walk with help after age 13 months, autonomous walk after age 18 months. Delayed language development was defined by bisyllabism after age 9 months, or first words after age 18 months. For one patient born pre-term (patient T2), the age for neurological development milestones was reported in corrected age—as she had to be compared with other patients born full-term. Neurological symptoms were defined by persistent axial hypotonia for toddlers, limb hypertonia (dystonic or spastic), dysphagia, neuropathic pain, VSGP or “oculomotor apraxia”, gelastic cataplexy and seizures. We considered as cerebellar signs: ataxia, dysmetria and/or impaired global or fine coordination and/or clumsiness. Neurological signs that were not specified as present or absent in the medical reports were classified as “data unknown”. The interpretations of cerebral MRIs were collected as reported in the medical files, MRIs were not re-read.

### Visceral manifestations

Visceral manifestations included: neonatal cholestasis, hepatomegaly, splenomegaly, cirrhosis and lung disease. The visceral signs were considered as absent if not mentioned in the medical file. We collected the date of onset and duration of jaundice; and we considered the clinical definition of visceromegaly and the age if it was noticed. Specific lung involvement included: recurrent bronchiolitis or asthma or chronic cough with interstitial pulmonary disease on chest X-ray and/or pulmonary infiltration with foam cells confirmed by bronchoalveolar lavage. Aspiration pneumonia and repetitive pulmonary infection were considered to be a consequence of neurological disease due to dysphagia and swallowing troubles. Some patients needed long-term oxygen therapy or non-invasive ventilation at home.

Weight and height curves were not available in most cases. The malnutrition was defined as a body mass index < 3rd percentile, or a clinical aspect of malnutrition mentioned in the observation. Patients who needed nutritional support had enteral feeding by long-term nasogastric tube, or gastrostomy.

### Treatment

In France, miglustat was available from 2006 in an early access programme for a limited number of 20 patients with NP-C, excluding those with an EI form. Miglustat did not become available to all NP-C patients with neurological symptoms until 2009, after market authorisation (MA).

Dosing was adjusted based on body surface area as recommended [2, 24]. The adjusted dose for children is around 300–400 mg/m^2^/day, beginning with 100 mg once a day for patients whose body surface area is ≤ 0.47 m^2^. Adverse events mentioned in the medical files were collected. Digestive adverse events were considered as severe if, despite long-term low disaccharide diet and/or symptomatic drugs, a decrease of miglustat dosing or a temporary stop was required. Data regarding associated medications (antiepileptic, antalgic, antispastic, antidystonic) prescribed at any time of follow-up were also collected.

### Data analyses

Age comparisons were studied using a Mann–Whitney test, whenever data were available for 8 patients or more in each group. Fisher’s exact tests were used for contingency analyses. We plotted the survival curves with a Kaplan–Meier analysis and compared these curves with a log-rank (Mantel-Cox) test.

## Results

### Patients

During the period 1990–2013, a total of 153 new patients with NP-C (from perinatal period to adulthood) were diagnosed in France, among which 32 patients (21%) with neurological onset before 2 years of age, who were thus classified as suffering from the EI neurological form of the disease. Eleven (7%) other patients diagnosed during the same time interval who had died between 3 days and 5.5 months of age from liver and/or respiratory failure, before neurological onset or with hypotonia that could be attributed to the severe systemic disease, were not included in the study, as they belonged to the systemic perinatal rapidly fatal form.

Among the 32 patients with EI-NP-C, medical files were unavailable for 4 of them; a 23 month-old patient with too short follow up of miglustat treatment (< 3 months) was excluded; and a further patient was not considered as an EI-NP-C form because his development before miglustat therapy more resembled that of a late-infantile form: independent walking was acquired at age 22 months, and remained stable at age 3 years 7 months, before miglustat onset. The investigated cohort (Tables 1 and 2) thus comprised 26 patients, 10 (38%) females and 16 (62%) males, who had been followed in 15 different French hospitals. There were two sibling pairs. The 10 treated patients (named T1 to T10) were born between 2006 and 2012. Among the 16 untreated patients (named U1 to U16), 15 were born between 1987 and 2005, and one (U7) in 2012. Four patients in the untreated group were lost to follow up. All others are now deceased. A filipin test was performed in all patients but one (a younger sibling), and invariably showed a classical pattern (massive lysosomal accumulation in perinuclear vesicles). All patients were fully genotyped: 24 patients (92%) had *NPC1* pathogenic variants, 2 (8%) *NPC2* pathogenic variants (Table 1). Although living in France, patients were of varied ethnic/geographic origin. About half of the patient’s families originated from other countries, including North Africa, Turkey, and Sri Lanka/South India. Individual clinical data for a number of parameters are given in Supplementary Table S1.

**Table 2:** Clinical characteristics of the untreated versus treated groups

	Non-miglustat group n = 16	Miglustat-treated group n = 10	p value
Year of birth			
1987–2005	15	0	
2006–2012	1	10	
Sex ratio ( boys/girls)	11/5	5/5	0.42
NP-C1 (*NPC1* gene)	14	10	0.51
NP-C2 (*NPC2* gene)	2	0	
Consanguinity	44% (7/16)	50% (5/10)	
Age at diagnosis (years)	1.88 y (antenatal-4.9)	0.3 y (0.04–1.4)	0.02
Age at neurological onset (months)	12 m (3–18)	9 m (5–18)	0.22
Diagnosis before neurological onset	37.5%	100%	0.003
Age at death (years)	11/16 4.42 (2.58–6.52)	10/10 5.56 (2.79–7.43)	0.16
Visceral signs			
Neonatal cholestasis	5/16 (31%)	9/10 (90%)	0.005
Duration (months)	3 m (3–6); uk n = 2	8 m (3–9); uk n = 3	Nd
Specific pulmonary dysfunction	5/12 (42%)	6/10 (60%)	0.70
Splenomegaly	13/14 (93%)	8/8 (100%)	> 0.99
Age at discovery (months)	1.4 m (0.01–28)	1 m (0.1–4)	0.20
Hepatomegaly	12/13 (92%)	9/9 (100%)	> 0.99
Age at discovery (months)	3.2 m (0.4–33)	1 m (0.1–4)	0.07
Nutrition			
Malnutrition at last follow-up	4/13 (31%)	2/8 (25%)	1
Nutritional support	8/9 (88%)	8/8 (100%)	> 0.99
Age at start (years)	2.75y (1.5–6)	2.92y (0.5–6.7)	0.92
Medications			
Oxygen or non-invasive ventilation	2/7 (29%)	7/9 (78%)	0.13
Antiepileptics	5/7 (71%)	4/8 (50%)	0.61
Antispastics or antidystonics	6/7 (86%)	7/10 (70%)	> 0.99
Antalgics	5/7 (71%)	6/7 (86%)	> 0.99

Percentages were calculated taking into account the number of patients for whom information was available. Results are reported as median (range). Abbreviations: y = years; m = months; uk = data unknown; nd = no statistical test due to inadequate sample size

### Genetic characteristics (Table 1)

Among variant *NPC1* alleles, a majority (56%) were missense, with a high (29%) proportion of null alleles. No p.(Ile1061Thr) nor p.(Pro1007Ala) allele was observed. The most recurrent variant (17% of alleles) was p.(Gly1240Arg), typically observed in patients originating from Sri Lanka/South India. An important number of patients carried a homozygous variant: 10/16 in the untreated group, 6/10 in the treated group. This was in large part related to a high rate of consanguinity (Table 2), present for the untreated group in 6/15 families, and for the treated group, in 5/10.

### Disease characteristics

#### Age at diagnosis and first neurological symptoms

**Fig. 1:**
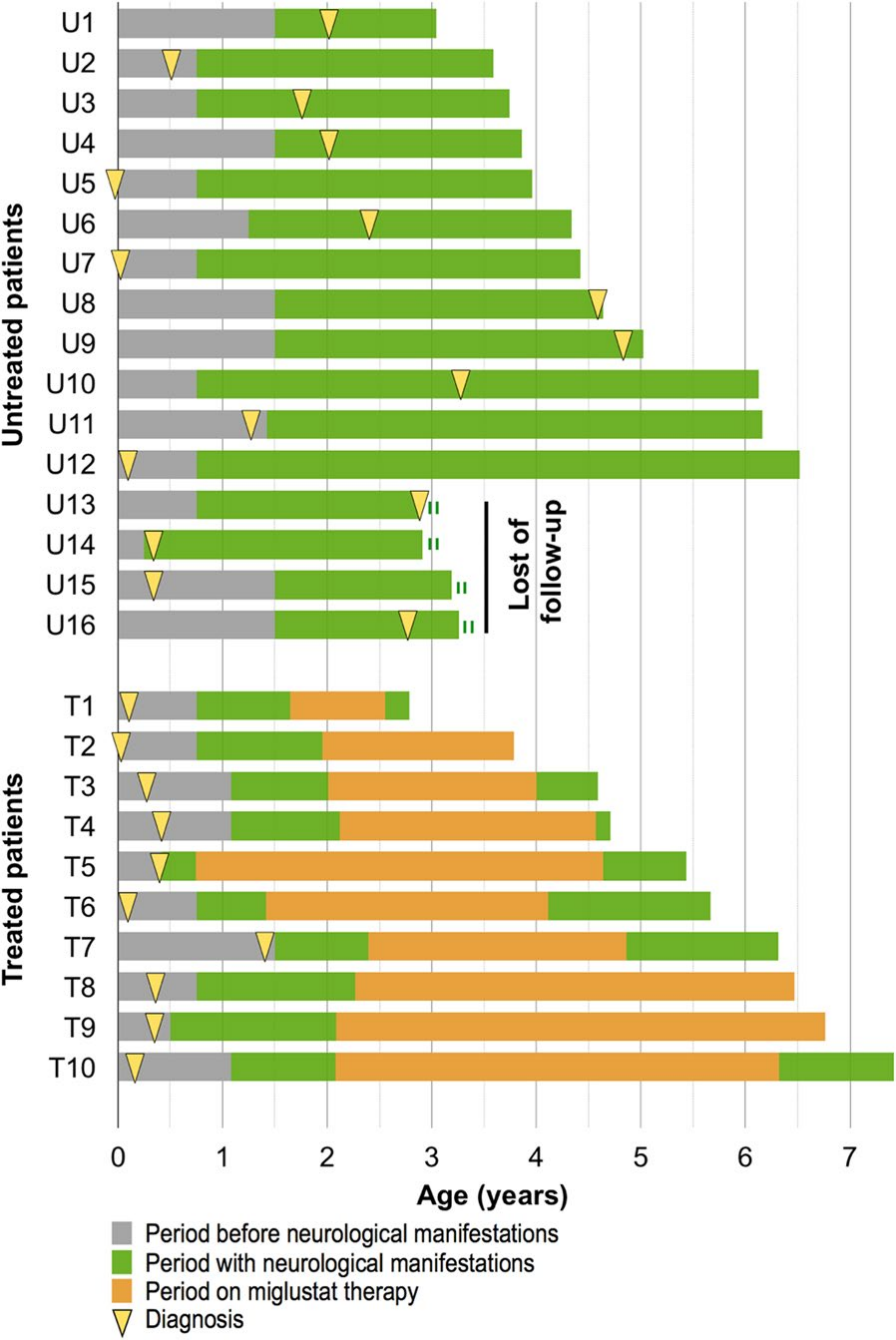
Timeline of neurological disease, diagnosis, miglustat therapy, and lifespan in the treated vs untreated groups

The age at neurological onset (defined as described in the Methods section) varied between 3 and 18 months and did not significantly differ between groups (Table 2; Fig. 1): the median age was 12 months in the untreated group (range 3–18) and 9 months (range 5–18) in the treated group (p = 0.22). The diagnosis of NP-C disease, however, was made significantly earlier in the treated group compared to the untreated one (median age at diagnosis 3.6 months (14 days to 17 months) and 22.5 months (ante-natal to 4.9 years), respectively; p = 0.02) (Table 2). The diagnosis was made before neurological manifestation for 16 patients, because of neonatal cholestasis and/or hepatosplenomegaly, or sibling history, with a significantly higher frequency in the treated group (100% versus 37.5%, p = 0.003) (Table 2; Fig. 1).

The psychomotor milestones and median (range) age for achievements in patients with available information are specified in Table 3. Hypotonia and motor development delay were the most frequent first neurological sign of NP-C disease (85% of all the 26 patients). More precisely, the first sign was: axial hypotonia for patients U14, T5 and T9 (at 3, 5 and 6 months of age, respectively); delayed autonomous sitting for 6 untreated patients and 4 treated patients; delayed walk with assistance for 3 treated patients; delayed autonomous gait for 5 untreated patients and 1 treated patient. Autonomous gait was noted as never acquired for 10 untreated and 9 treated patients (missing information for 3 patients). Ataxia and psychomotor regression constituted the first reported neurological sign for only 15% of the patients, all in the untreated group: U13 acquired standing position with assistance and rapidly lost it at 9 months; U16 lost ability to stand following a bronchitis at 18 months; ataxia was noticed at the age of 15 and 17 months for patients U6 and U11.

**Table 3:** Motor milestones and neurological symptoms of NP-C in the untreated vs treated groups (in chronological order)

	No-miglustat group n = 16	Miglustat treated group n = 10	p value
Autonomous sitting position			
Age at acquisition	0.75 (0.5–2) uk = 4	0.83 (0.7–1.4) na = 1, uk = 2	x
Age at loss	2.83 (2.25–5.9) uk = 6	3.1 (1.9–3.8) uk = 1	0.8
Standing position with help or walk with help			
Age at acquisition	1.25 (0.75–2) uk = 5	1.25 (0.8–2.25) na = 3	x
Age at loss	2.5 (1.5–5.5) uk = 9	2.91 (2.4–3.4) uk = 1	x
Autonomous walk	1.7 (0.8–2) na = 10, uk = 2	na = 9, uk = 1	x
Delayed language development	1.63 (0.75–2.3) uk = 10	1.56 (0.75–2.4) uk = 2	x
Cerebellar signs	1.88 (1.25–3.5) uk = 7	2.21 (1.2–3.9) uk = 2	0.60
VSGP or oculomotor apraxia	2.33 (1.6–5.25) uk = 11	2.58 (0.7–4.6) uk = 1	x
Limb hypertonia	2.54 (2.1–4.4) uk = 6	2 .13 (0.7–3.9) uk = 2	0.20
Seizures	2.75 (2.4–4.5) uk = 8	3.37 (2.75–5.6) uk = 6	x
Neuropathic pain	2.83 (2.75–4) uk = 12	3.08 (0.7–4.8) uk = 5	x
Swallowing trouble	3.04 (0.7–4.6) uk = 8	2.37 (0.25–2.8) uk = 4	x
Gelastic cataplexy	3.5 (2.2–4.5) uk = 13	4.08 (2.5–4.6) uk = 6	x

The ages are reported as median (range) and expressed in years. Ataxia, dysmetria and/or impaired global or fine coordination were considered as cerebellar signs. Limb hypertonia reported here was spastic or dystonic. Abbreviations: x: no statistical test due to inadequate sample size; uk: data unknown (= n); na: never acquired (= n); VSGP = vertical supranuclear gaze palsy

#### Clinical symptoms and evolution

Many data were missing in the later follow-up of patients (Table 3) but by order of frequency, neurological manifestations included: swallowing troubles (18 patients, 16 of whom required enteral feeding by nasogastric tube or gastrostomy, all before 3 years of age), limb hypertonia (18 patients), cerebellar signs (16 patients), VSGP (14 patients), seizures (12 patients), neuropathic pain (9 patients), gelastic cataplexy (7 patients). Regarding visceral symptoms, medical records mentioned hepatomegaly and/or splenomegaly for 92% of all patients (during neonatal period for 50% of them), a pulmonary dysfunction for 77% of all patients, and a history of neonatal jaundice for 54% of them. Except for neonatal cholestasis, there was no statistical difference for visceral symptoms and nutritional support between the two groups (Table 2). Lung involvement could be classified as specific of NP-C for 11 (42%) patients with documented interstitial pulmonary disease on chest X-ray; 3 of them had pulmonary infiltration with foam cells confirmed by bronchoalveolar lavage. Pulmonary disease was non-specific for 9 (35%) patients. Need of long-term oxygen therapy was reported for 9 (35%) patients, among whom 6 had specific pulmonary disease. Of note, oxygen or non-invasive ventilation had been more frequently used in the miglustat-treated group (78%) than in the non-treated group (29%).

The global median age at death, known in 22/26 patients, was 56 months (range 33–89 months).

#### Brain imaging

Results of cerebral MRI were available for 15 patients (6/16 untreated ones, 9/10 treated ones). The first MRI was performed at a median (range) age of 1.9 years (0.4–4.8). Four patients had a normal first MRI at 5 m (T2), 8 m (T8), 22 m (U11), 31 m (T6). Two of these had a later MRI during follow-up, which became abnormal: T2 at 2y of age, on miglustat, showing delayed myelination: and T8 at 2y3m of age, before miglustat treatment, showing abnormal periventricular white matter and atrophy of the subcortical regions. Observed radiological abnormalities, eventually associated in the same patient were: external hydrocephaly for 3 patients (at 7 m, 23 m, and 27 m); abnormal white matter signal (delayed myelination or demyelination for 12 patients (7 treated ones, 5 untreated ones), on MRI performed after or at 12 months of age); atrophy of the corpus callosum and/or the periventricular white matter and/or the sub-cortical regions for 4 patients, on MRI performed after or at 20 months of age; atrophy of the cerebellar vermis, at 25 months of age for one patient.

### Miglustat therapy

#### Miglustat therapy characteristics

Ten patients (38%) were treated with miglustat since February 2009, as it was not available for EI-NP-C patients in France before MA. This explains a longer delay between neurological onset and treatment for the 6 patients diagnosed before 2009 (from 10 to 19 months) compared to less than 7 months for the 2 patients diagnosed in 2012 and 2013. No EI-NP-C patient of our series was treated with miglustat before neurological onset: this follows international recommendations, and our observation of patients diagnosed on visceral symptoms during the perinatal period or before 1 year of age, showing no neurological signs in late adolescence or adulthood [case 33 in (5) and a case discussed in (25) currently 30 year-old, or with neurological onset in adolescence [case 6 in (25)]. Characteristics of miglustat therapy are summarized in Table 4 and individual timelines shown in Fig. 1. The median age at miglustat start was 24.5 months, ranging from 17 to 29 months, except for patient T5, treated from the age of 9 months. The median duration of treatment was 30 months (range 11–56). The median initial dose (reached after a progressive introduction except for one patient) was 265 (70–690) mg/m^2^/day, and the median maximal dose 355 (210–690) mg/m^2^/day. The recommended doses of miglustat were respected until the end of therapy for 5 patients, lowered for 3, and unknown for 2 of them. Table 1 lists the associated therapies prescribed at any time of evolution, which showed no significant difference between the two groups.

**Table 4:** Characteristics of miglustat therapy in the treated group

Patient	Delay between neurological onset and start of miglustat	Age at start of miglustat	Duration of miglustat	Continuous miglustat therapy	Dose at onset	Maximal dose	End-dose
T1	11 m	20 m	11 m	Yes	690	690	425
T2	14 m	23 m	22 m	Yes	205	410	uk
T3	11 m	2 y	24 m	Yes	210	210	160
T4	10 m	2 y 1 m	2 y 5 m	Temporary (1 m) discontinuation	280	350	145
T5	4 m	9 m	3 y 11 m	Yes	280	360	170
T6	8 m	17 m	2 y 8 m	Temporary (2 m) discontinuation	250	360	340
T7	11 m	2 y 5 m	2 y 6 m	Yes	70	350	300
T8	18 m	2 y 3 m	4 y 2 m	Yes	110	320	uk
T9	19 m	2 y 1 m	4 y 8 m	Yes	460	640	230
T10	12 m	2 y 1 m	4 y 3 m	Yes	290	350	330

Doses are expressed in mg/m^2^/day. End-dose corresponds to the dose before death, or at the stop of miglustat, or at last follow-up

*y* years, *m* months, *uk* data unknown

#### Tolerability

Gastrointestinal (GI) adverse events (diarrhoea, anorexia, weight loss, failure to thrive, vomiting, abdominal bloating) were reported in 7 patients (70%). Five patients (50%) had severe GI adverse events, requiring a decrease of miglustat dosing or a temporary stop, despite long-term low disaccharide diet and/or symptomatic drugs. Two (20%) patients had to stop miglustat temporarily: patient T6 stopped treatment during 2 months because of vomiting; patient T4 had miglustat introduction at full dose and had to stop treatment during 1 month at the beginning because of anorexia, flatulence and diarrhoea. Tremor and low platelets were reported in 2 patients (20%). Seven patients (70%) stopped miglustat therapy before death because of neurological worsening, 4 of them had also associated adverse events (3 patients with digestive symptoms, one patient with mild thrombocytopenia (platelets = 120 × 10^9^/L).

#### Effects of miglustat treatment on neurological manifestations

All 10 treated patients worsened during miglustat therapy regarding loss of psychomotor acquisitions or new neurological symptom, as shown in Table 3. However, 6 patients (60%) had shown an initial improvement. Patient T5 who started miglustat when 9-month-old (much earlier than all others) improved during the first 18 months of treatment, showing acquisition of sitting position at 11 months, improvement of VSGP, first words at 21 months, walking with assistance at 27 months, but she lost the latter ability at 33 months. Patient T3 (miglustat start when 24 month-old) improved after 3 months of treatment, with more stable sitting station, emergence of babbling, less tremor. Patients T6 and T7 (miglustat start at 17 months and 29 months, respectively) showed improved alertness, and patient T6 also acquired standing position at 18 months. Patient T10 (miglustat start at 25 months) had better tonus and communication, he could associate two words at 27 months.

**Fig. 2:**
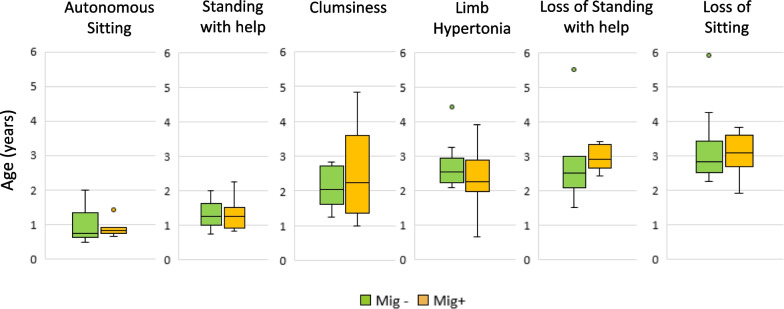
Comparative ages for acquisition/loss of motor milestones and onset of some neurological symptoms. *Mig*+ miglustat-treated patients, *Mig− *untreated patients

Nevertheless, the untreated patients had also shown some new psychomotor acquisitions in the first two years of life (Table 3), and globally, there was no difference between the two groups. A graphical comparison of known ages for acquisition/loss of major motor milestones and onset of some neurological symptoms for miglustat-treated vs untreated patients is also given in Fig. 2.

#### Effect of miglustat therapy on survival

**Fig. 3:**
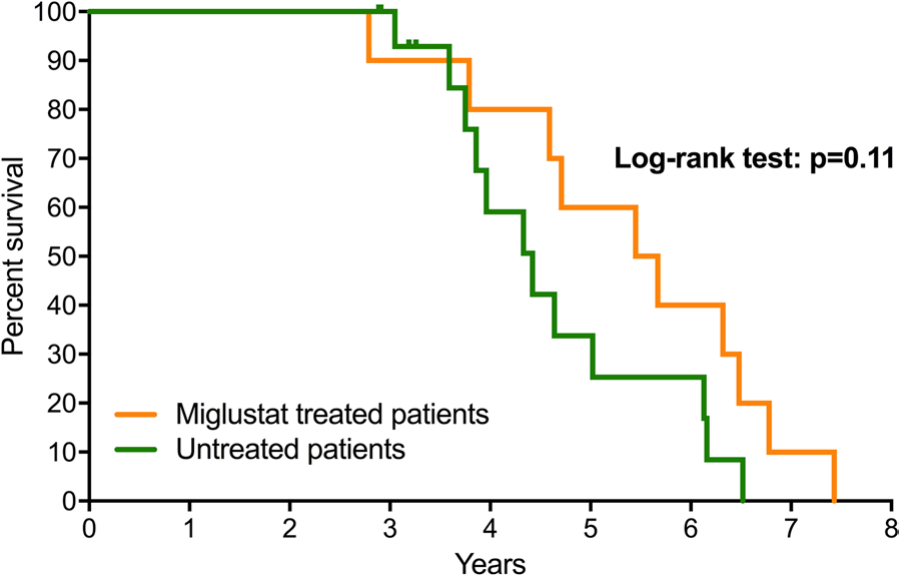
Comparative Kaplan–Meier survival curves in miglustat-treated or untreated patients. Median survival age: 4.42 years for the untreated group; 5.56 for the treated group

In the untreated group, the median survival was 4.42 years, with an age at death comprised between 3.1 and 6.5 years for 12 patients; 4 patients were unfortunately lost to follow-up when aged 2.9 to 3.3 years. In the treated group, the median survival was 5.56 years (age at death ranging between 2.8 and 7.4 years; n = 10). Figure 3 shows the Kaplan–Meier survival curves, which are not significantly different (Log-rank test: p = 0.11). No patient was alive beyond the age of 7 years 5 months in either group. Of note, patients U7 and T1 were brothers; T1, treated with miglustat, had died at the age of 2 years 9 months because of severe pulmonary involvement with long-term oxygen therapy, whereas his untreated younger brother died at the age of 4 years 5 months with a similar neurological involvement, but no specific pulmonary disease.

## Discussion

In the global French NP-C cohort diagnosed between 1990 and 2013, the EI-NP-C form constituted globally 21% of cases, a proportion very similar to that (22%) reported for Italy, but higher than for Czech Republic and above all the UK, with 15% and 5%, respectively [8, 26–28]. Among the 10 patients with *NPC2* mutations diagnosed during this period, only 2 developed an EI-NP-C form.

By reference to a recent literature survey [26], the present French series of 26 well characterized patients constitutes the largest EI-NP-C cohort with data on psychomotor achievements, neurological outcome and survival. It is also the first study to evaluate miglustat efficacy on EI patients by comparison to an untreated group.

Cell biology studies in cultured fibroblasts, primarily used to confirm the diagnosis, disclosed in all cases (with either *NPC1* or *NPC2* mutations) a profound alteration of LDL-cholesterol cellular trafficking, with massive accumulation of unesterified cholesterol in lysosomes, and in all cases studied, a severe block of LDL-induced cholesteryl ester formation (classical biochemical pattern) [20]. This profile is in good accordance with results in EI-NP-C patients published from Czech Republic [28], Italy [27], and Spain [29, 30]. A correlation between two null alleles and the EI-NP-C form is usual [3, 26]. Our data confirm that the missense *NPC1* variants p.(Arg518Gln) and p.(Thr1036Met) are severe [26], and further pinpoint p.(Ala172Pro) and p.(Gly1240Arg) as very deleterious, correlating with an EI-NP-C form when homozygous or associated with another severe allele. Of note, the c.1553G>A variant leading to the p.(Arg518Gln) missense is known to also cause a splicing error with exon 9 skipping [31]. Previous functional studies on the *NPC2* p.(Ser67Pro) and p.(Cys99Arg) variants had shown that these mutated proteins were misfolded and retained in the endoplasmic reticulum [32].

Since miglustat was only approved in 2009, treated patients were born and diagnosed in a more recent period than those in the untreated group. This may explain some observed differences between groups not linked to the treatment (Table 2). Under-reporting of neonatal cholestasis in the untreated group cannot be excluded, as a retrospective history of neonatal cholestasis, if not severe, could have been neglected in older medical reports. On the other hand, in recent years NP-C testing in neonatal cholestasis has been performed in France nearly systematically after exclusion of the most common causes [5], a factor which could also contribute to the younger age at diagnosis observed in the treated group. Furthermore, availability of a specific treatment has led to a better awareness of NP-C disease and to a closer clinical monitoring. The observation that all patients were diagnosed before neurological onset in the treated group (100%), compared to only 37.5% in the untreated group (p = 0.003) is likely a consequence of these combined factors. Of note, the strategy for laboratory testing had remained similar for both groups, since plasma biomarkers [3, 33] were not routinely introduced in France before 2015.

Inclusion criteria about age of neurological onset, steps of psychomotor development and neurological progression/outcome were clearly defined. Importantly, we chose to exclude isolated hypotonia as initial neurological symptom when it was observed in infants with a poor general state, i.e. life threatening hepatic or respiratory disease, because in our experience it does not necessarily reflects the subsequent neurological form. Categorization of a patient as EI-NP-C form on this sole criterion likely explains rare outliers with survival into adult age in NP-C registries [9].

Our clinical observations are consistent with the comprehensive review on EI-NP-C published by Seker Yilmaz et al. [26]. Neonatal cholestasis of variable length had been present in 54% of the cases, hepatosplenomegaly was nearly constant, and specific pulmonary dysfunction had occurred in about half of them. All patients in the present EI-NP-C series began neurological symptoms before 18 months of age (median global age of 9 months, slightly earlier than in [26]; only few patients temporarily acquired autonomous walking, and none did acquire a fully normal gait. Hypotonia and motor development delay were the first neurological sign in 85% of the patients, ataxia, and psychomotor regression in the remaining 15%.

No record of VSGP was found for 11 of the 16 untreated patients, but this sign was noticed in all patients but one in the more closely monitored treated group, indicating that it also belongs to the EI-NP-C form, although not an early sign (observed at a median age of 2.3–2.5 years). During the later course of disease, about half of the patients developed seizures, and approximately one fourth gelastic cataplexy. Limb hypertonia reported during the course or EI-NP-C contrasted with early hypotonia and combined spasticity with pyramidal signs and rigidity due to dystonia. Lower limbs were more severely involved than upper limbs, and medications were poorly efficient using antispastic, antidystonic and antalgic drugs in more than 75% of patients. As for later onset NP-C forms, swallowing troubles were very frequent in EI-NP-C patients, with a need of nutritional support for 88% of untreated patients and 100% of miglustat treated patients, at a similar median age of 2.75 and 2.92 years respectively.

In our study, the median age at death was 4.4 years in the untreated group (4.7 years for all patients), compared to 4.0 years in the survey by Seker-Yilmaz et al. [26], with also a narrower range (2.8–7.4 years) in our cohort.

Because of the retrospective nature of the study, information bias (data not reported in medical files, subjective interpretation of data) might have involved even data on neurological manifestations. It must be underscored that for many parameters, the small size of samples and the amount of unknown items prevented statistical treatment. Finally, we did not attempt to evaluate the progression of functional disability scores in our individual patients. Several NP-C severity scales, in which a composite score is calculated after assessment of 4 or 5 key neurological domains (ambulation, fine motor skills, language, swallowing, cognition) or more, have been developed [17, 34–37] and used in natural history studies and evaluation of therapies [10, 15, 17]. In this study, they could not be used for retrospective untreated patients. Furthermore, except for the more recent one [37], these validated scales do not include milestones before ambulation, and therefore—although they were used in some studies involving patients with the EI form [10, 17–19]—, none of them was at the time of our study fully suitable for precise evaluation of toddlers, as would have been needed here.

Notwithstanding the above limitations of this retrospective study, assessment of key parameters of neurological disease progression indicated no significant long-term improvement for patients with EI neurological onset who had received miglustat therapy for a median period of 30 months. Among others, the present study completes, 3.9 years later, the follow-up for 6 of the early-infantile NP-C patients included in our 2012 early study [10] (see method section), in which the median duration of miglustat therapy was only 1.3 years. Data at that time already indicated a global poor response to miglustat. Héron et al*.* [10], however, suggested that a short interval between neurological involvement onset and the start of miglustat therapy and/or young age at treatment start could be associated with a better outcome of therapy, at least initially, as observed in case #2 (same as #T5 in our study) with the shortest delay (4 months) between neurological onset and miglustat initiation. This better initial outcome was confirmed and slightly extended in the present study, but it was not maintained, and the patient died at 5 years 5 months of age. Several other publications reported a transient stabilization or improvement of EI patients during the first months (or years) of miglustat therapy, especially when the treatment was started early, but these studies either described no change in long-term outcome [38, 39], or the follow-up was too short to conclude on long-term outcome [17, 40, 41]. The results of the retrospective observational chart review study by Pineda et al. [42] cannot be compared to our study, because early infantile and late infantile patients were analysed together (mean age at first neurological symptom was 2.28 years), and the control group was much smaller than ours. Finally, initial results of an international NP-C registry [18] showed improved or stable neurological disease in 33% of patients with the EI form who had received continuous miglustat therapy for an average follow-up period of 1.8 years, compared with 50% in the late-infantile group and 79% in the juvenile group. In the final report of this prospective registry [19], results in the 19 treated EI-NP-C patients confirmed the lack of significant stabilization of the composite disability score, with a mean at enrolment of 0.59 and 0.70 at last follow-up. Our present results are thus globally in line with most published studies, including our own [10], showing that while miglustat can stabilize neurological manifestations in later onset forms of NP-C, its long-term efficacy in the EI form has so far not been clearly demonstrated.

Regarding the specific question of survival, our results suggested a trend towards treatment efficacy, which, however, was not significant. A role of potential better global care in the treated group (covering the more recent period 2007–2017) cannot be fully excluded, as oxygen or non-invasive ventilation was more frequently used in our miglustat treated group (78%)—with date of birth after 2006 for all patients—, than in the historic non treated group (29%)—date of birth before 2006 for all but one—. Similarly, use of nutritional support for 100% of EI patients in the miglustat-treated group reflects earlier indication of nutritional support in more recent patients, to improve general state and comfort. A recent extensive survival study on 333 patients with NP-C (eventual therapy unknown) who had died between 1981 and 2018 [43] has concluded that supportive medical care had not impacted survival in the recent past. However, since no stratification by clinical form could be done, a slight change such as that observed by us could hardly have been captured.

In any case, treated or not by miglustat, no EI-NP-C patient in our series was still alive beyond 7 years 5 months. Our observation on survival is consistent with the first comparative international survival study recently published by Patterson et al. [23], which among others includes data from the French cohort. A statistically significant reduction in risk of mortality for miglustat-treated patients was observed in the overall analysis. In clinical subgroups analysis, however, a significant level was only achieved for the late infantile patients, and the smallest improvement of survival was found in the miglustat-treated EI-NP-C group.

## Conclusion

Our data indicated that benefits of miglustat in monotherapy on neurological disease of patients with EI-NP-C were very small and not sustained—if briefly present—. We further observed no significant beneficial effect of miglustat on survival in this particularly severe form of the disease However, the long lag phase between first neurological symptoms and start of miglustat therapy for many of our patients, diagnosed before treatment availability, needs to be taken into consideration.

A subsidiary benefit of this retrospective study was to add further knowledge on the longitudinal course of EI-NP-C in a fairly homogenous cohort. Analysis of the data emphasized the need to develop an EI-NP-C-specific disability scale. Collecting in prospective patients more precise neuro-developmental data is becoming of particular importance, since new therapeutic options, including combined therapies, will become available for patients with EI-NP-C, with hopefully a better effect on the disease course.

## Supplementary Information

**Additional file 1. Table S1**: Clinical characteristics of patients with EI-NP-C.

## Data Availability

The dataset analysed during the current study is not publicly available due to patients’ privacy issues, but is available from the corresponding author on reasonable request.

## Abbreviations

CETNL: National Committee for Evaluation of Treatment of Neurolipidoses
CRML: Reference Centre for Lysosomal Diseases
EI: Early infantile
MA: Market Authorization
MRI: Magnetic resonance imaging
NP-C: Niemann-Pick disease type C
VSGP: Vertical Supranuclear Gaze Palsy
